# On Your Mark, Get Set, Choose! A Randomized Cross-Over Study Comparing Fixed and Self-Selected Rest Periods in Interval Running Among Professional Female Soccer Players

**DOI:** 10.1186/s40798-024-00803-8

**Published:** 2025-01-14

**Authors:** Asaf Ben-Ari, Yedidya Silverman, Uri Obolski, Israel Halperin

**Affiliations:** 1 Department of Health Promotion, Faculty of Medical and Health Sciences, School of Public Health, Tel-Aviv University, Tel-Aviv, Israel; 2 Sylvan Adams Sports Institute, Tel Aviv University, Tel-Aviv, Israel; 3 Department of Epidemiology and Preventive Medicine, Faculty of Medical and Health Sciences, School of Public Health, Tel-Aviv University, Tel-Aviv, Israel

**Keywords:** Autonomy, Self-selected rest, HIIT, Soccer

## Abstract

**Background:**

Studies on rest durations during high-intensity interval training (HIIT) often compare fixed and self-selected (SS) rest allocation approaches. Frequently, the rest duration under SS conditions is unlimited, leading to inconsistent total rest durations compared to fixed rest conditions. To address this limitation, we recently compared fixed and SS rest conditions during cycling HIIT sessions, while keeping the total rest duration equivalent. However, our protocol required athletes to divide a long total rest duration (720 s) across nine intervals, which may have been overly cognitively demanding. The current study aimed to explore the effects of the SS approach with a simplified rest allocation task on performance, physiological, and psychological outcomes.

**Methods:**

Following a familiarization session, 24 professional female soccer players completed two running HIIT sessions on a non-motorized treadmill. Each session consisted of twelve 15 s intervals, divided into three blocks, with the goal of maximizing the distance covered. In both conditions, the between-interval rest duration per block amounted to 270 s. In the fixed condition, the rest was uniformly allocated to 90 s between each interval, whereas in the SS condition, the athletes chose how to allocate the entirety of the 270 s of rest. We compared the following outcomes: distance, heart rate, perception of fatigue, effort, autonomy, enjoyment, boredom, and athletes’ preferences. Outcomes were compared using aggregated measures via paired univariate tests, and across the intervals via mixed-effects models.

**Results:**

We observed comparable results in most outcomes with the exception of higher autonomy (1–15 points) in the SS condition (mean difference = 2.1, 95%CI (0.9, 3.3) points) and a negligibly higher heart rate in the SS condition when comparing the observations across intervals (estimate = 2.5, 95%CI (0.9, 4.2) beats × min^−1^). Additionally, participants chose to rest for longer durations as the block progressed. Finally, the majority of participants (65%) favored the SS condition.

**Conclusion:**

This study further solidifies that SS and fixed approaches with matched total rest durations result in similar performance, physiological, and psychological responses. This effect persists even when the total rest duration required to be allocated is relatively short. Therefore, coaches and trainees can choose either approach based on their preferences and training goals.

**Supplementary Information:**

The online version contains supplementary material available at 10.1186/s40798-024-00803-8.

## Key Points

- Most studies comparing fixed and self-selected (SS) rests in HIIT allowed participants in the SS condition to rest for as long as they wanted, thereby not matching total rest duration.
- This unlimited SS rest approach impairs the ability to differentiate the effects of choice from those of rest duration on the various outcomes.
- Here, we compared the effects of fixed and SS rests in HIIT with matched total rest duration using a non-motorized treadmill.
- Outcomes in both conditions were similar, implying either method can be adopted depending on training objectives and athletes’ preferences.

**Supplementary Information:**

The online version contains supplementary material available at 10.1186/s40798-024-00803-8.

## Background

Soccer requires a highly developed aerobic capacity [1, 2]. Various training methods have been developed to help players build and maintain this capacity, with high-intensity interval training (HIIT) being particularly effective and time-efficient [3–5]. Numerous training types fit the HIIT definition, all sharing a similar structure: high-intensity work bouts interspersed with rest periods [6, 7]. In HIIT, the ratio between work and rest is one of the key factors determining the overall training effect [8–10]. While rest periods alleviate fatigue and enable subsequent efforts, they simultaneously decrease the aerobic stimulus of the session [9]. Therefore, selecting appropriate rest durations in HIIT sessions is crucial for personalizing training and optimizing training adaptations.

The most common approach to prescribing rest periods in HIIT involves fixed, predetermined durations with work-to-rest ratios ranging from 1:0.5 to 1:20 [7, 11]. While this method is convenient and efficient [3, 12], it does not account for individual physiological and psychological differences. For example, differences in fatigue accumulation, which in turn likely result in different durations required to recover [13]. An alternative is the self-selected (SS) approach, where athletes determine the duration of their rests. The SS approach offers several unique benefits. First, choice provision promotes one’s perceptions of autonomy [14] and enhances motivation [15] and enjoyment [16], all of which have an important role in competitive sports [17, 18]. Second, the SS method may better accommodate individual differences by allowing athletes to tailor rest durations to their current physiological and psychological readiness and anticipated performance [19]. Finally, the SS approach introduces a decision-making component that is relevant to many sports. For example, soccer players continuously make decisions in dynamic, unpredictable environments while considering their chances of success and their limited resources [20, 21]. The SS approach may enhance athletes' ability to adjust rest duration based on factors like remaining game time, score, and their physical and mental state.

Studies comparing the acute effects of fixed and SS rest durations during HIIT sessions have presented mixed results [19, 22–28]. Some have reported enhanced performance and psychological outcomes with SS rest periods [22–24], while others have shown the opposite [26, 27]. The significant variability in prescribed HIIT protocols (e.g., 4 × 4 min [24] vs. 12 × 30 m [22]) and the duration of rests in the fixed condition (e.g., 3 min [28] vs. 30 s [23]) may have contributed to these inconsistent results. However, these studies share a major limitation—the total rest duration between fixed and SS conditions was not matched. In the SS condition, participants selected their rests with no lower or upper limit, resulting in different total rest durations than those in the fixed conditions and, consequently, different training stimuli. This limitation makes it challenging to untangle the effects of choice from those of rest duration.

To overcome the latter limitation, we recently conducted a study employing a novel approach that matches total rest duration between conditions [14]. In that study, 24 male amateur cyclists performed two HIIT sessions in a crossover design consisting of nine 30 s cycling intervals. Under the fixed condition, participants rested for 90 s between intervals, thus accumulating 720 s of rest (8 × 90 s = 720 s). Under the SS condition, participants self-selected their rest durations out of the 720 s provided to them, which had to be fully utilized by the final interval. That means, participants self-selected how to allocate a total rest duration to each rest period rather than to self-select each rest period's duration without restrictions. All performance, physiological, and psychological outcomes were similar except for greater perceived autonomy in the SS condition. Furthermore, participants in the SS condition chose shorter rest durations in the first half of the session than in the second half, with some participants ending up with very long rest periods in the final interval (e.g., 3.5–4 min). We speculate that this resulted from participants finding it cognitively challenging to effectively distribute the 720 s of total rest across intervals, leading to miscalculations in rest allocation.

Given the above, the present study aimed to build on and expand upon our previous research by exploring the SS approach with three key modifications. First, we simplified the task of rest duration allocation to streamline the decision-making process for participants. Second, we tested the SS approach among female soccer players to broaden our understanding of its applicability across genders, as female athletes are underrepresented in sports science [29]. Lastly, we implemented the SS approach within a running HIIT protocol to align with the training modalities commonly used in soccer. We hypothesized that, compared to the fixed condition, the SS condition would optimize recovery, resulting in improved performance and higher perceptions of autonomy and enjoyment.

## Methods

### Participants

We recruited 24 professional female soccer players from various teams competing in the Israeli Women's Soccer First League (Tier 3 [30]). Inclusion criteria included healthy participants, aged between 16 and 45, with at least one year of professional-level soccer playing experience. Exclusion criteria included acute injury in the past 2 weeks, pregnancy, or being fewer than 6 months after childbirth. Table 1 summarizes the participants’ characteristics, including training history and weekly training volume. Recruitment was done through advertisements on various social media channels and by contacting teams from the Israeli women's soccer first league.

**Table 1:** Participants characteristics

Characteristic	N = 24^1^
Age [yrs]	22.0 ± 3.8 (16.9–28.9)
Height [cm]	163.4 ± 6.5 (151.0–175.0)
Weight [kg]	59.5 ± 5.9 (46.3–70.7)
Fat [%]	22.2 ± 4.8 (14.0–32.6)
Experience [yrs]	4.9 ± 2.9 (1.0–10.0)
Soccer training [sessions per week]	4.9 ± 1.1 (3.0–7.0)

^1^All values are presented as mean ± SD (range)

### Procedures

We implemented a within-participant, randomly assigned crossover design. All participants attended three laboratory sessions: a familiarization session and two experimental sessions. Given that menstruation may influence both performance and perceived performance [31, 32], we asked participants about their menstrual phase (“When was the first day of your last menstruation?”) and avoided scheduling any of the sessions during their menstruation phase. In addition, regardless of the menstrual cycle phase, we rescheduled sessions if a participant reported any menstrual cycle-related symptoms.

**Fig. 1:**
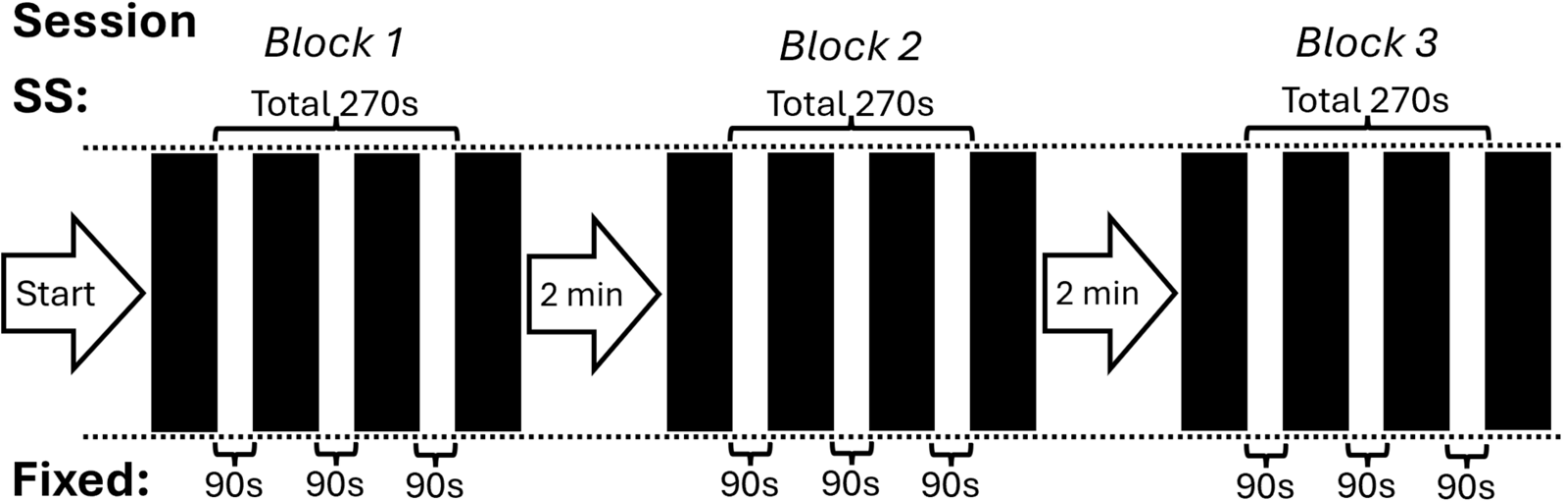
A diagram of the sessions’ protocol. Each black rectangle symbolizes an interval. The duration of between-interval rests in each condition are presented above (SS) and below (Fixed) the rectangles. SS = Self-selected

The HIIT protocol in the two experimental sessions consisted of twelve 15 s intervals performed on a non-motorized treadmill (Woodway^©^ Curve 3.0 Treadmill, Waukesha, United States). We considered several options for the duration of the intervals. Initially, we aimed to use 5–10 s intervals, which are commonly used in soccer training [3]. However, after conducting pilot sessions, we concluded that these intervals were likely too short to detect performance differences between conditions, particularly due to the relatively long acceleration time required on the non-motorized treadmill. Ultimately, we decided on 15 s as an optimal compromise between these competing factors. The twelve intervals were divided into three blocks of four intervals, with two minutes of rest between blocks, to simplify the task of time allocation under the SS condition. The two sessions only differed in the rest durations between intervals. Under the fixed condition, participants rested for 90 s between intervals, totaling 270 s of rest per block. Under the SS condition, participants selected how long they would rest between intervals. However, we matched the total rest duration between conditions, meaning that participants had to fully utilize 270 s of rest over the three rest periods of each block (Fig. 1 shows a diagram of the session’s protocol). We considered several options for the total rest duration in the SS condition. Following the pilot sessions, we ultimately chose 270 s as it proved long enough to allow participants to adjust their rest durations but was not excessively long, thereby minimizing unnecessary cognitive demands in managing rest distribution.

At the beginning of each session, we told participants their goal was to cover as much distance as possible across all intervals. Under the SS condition, we added that they should allocate their rest durations with this goal in mind. In both sessions, participants were allowed to choose whether to stand or walk during the rest periods except for the five seconds before the subsequent interval in which they were asked to stand still.

**Fig. 2:**
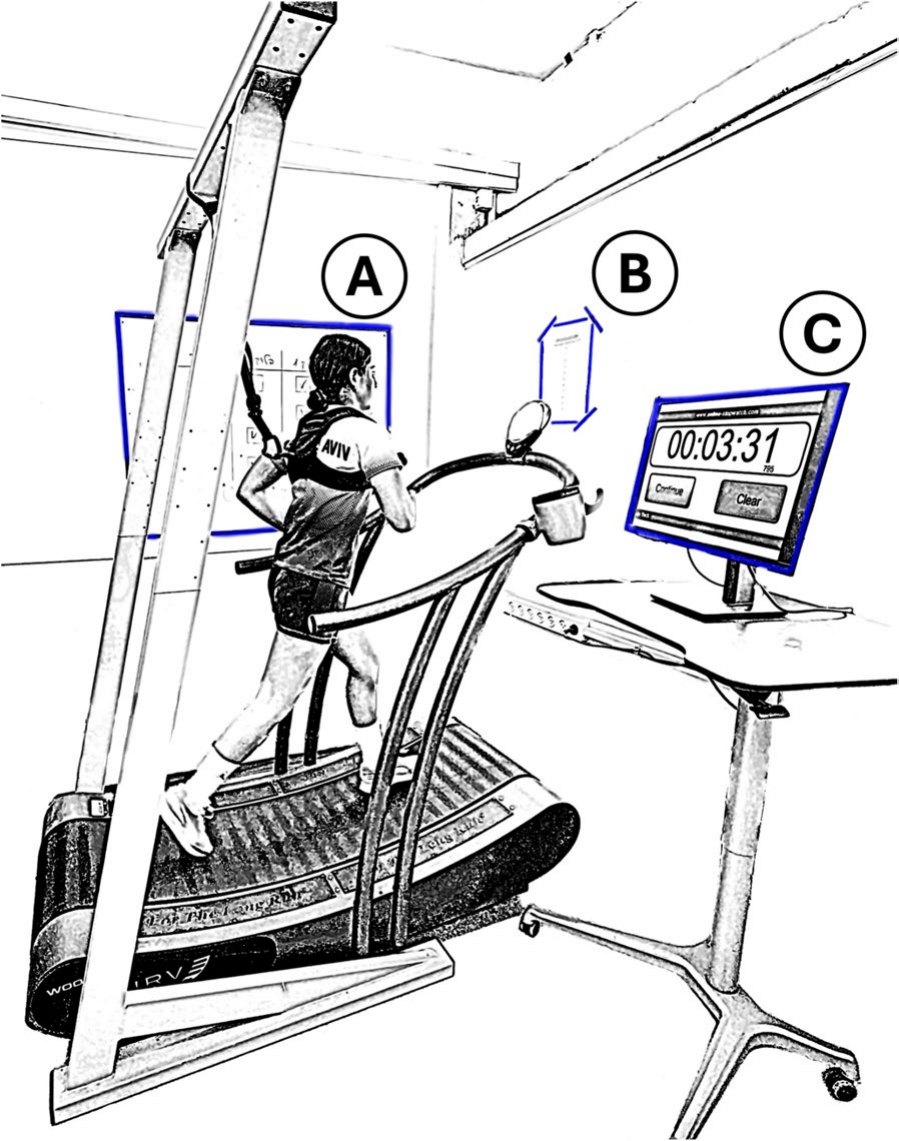
Experimental setup. **A** whiteboard with a list of completed intervals, **B** poster with a Rating of Perceived Effort scale, **C** computer screen with a timer

We provided participants with feedback regarding the remaining rest duration using a screen with a timer next to the treadmill. In the fixed-rest condition, the timer counted down from 90 s for each between-interval rest period. In the SS condition, the timer counted down from 270 s for each block (indicating the total rest duration still available to them). Once an interval was completed, the countdown started. When participants announced they were ready to start the next interval, the researcher began a 5 s verbal countdown, after which the interval would commence, and the timer was paused. Figure 2 shows the experimental setup.

All sessions began with the same general ~ 8 min warm-up that consisted of 2–3 min of light aerobic exercises (high knees, heel flicks, and jumping jacks), 3 sets of body-weight resistance exercises (air-squats and push-ups), and 2 min of self-selected dynamic stretches. After this warm-up, participants performed a baseline countermovement jump (CMJ) test. Next, participants performed an exercise-specific warm-up consisting of three minutes of easy steady-state running followed by five 10 s intervals at a gradually increasing speed. The intervals' speed corresponded to 40%, 60%, 80%, and two 100% of the participants' perceived maximal speed, interspersed with rest periods of 1–2 min. Two minutes after completing the exercise-specific warm-up, the first interval of the protocol began. Throughout the sessions, we recorded participants’ heart rate (HR), and immediately after each interval, we collected their ratings of perceived effort (RPE). Two minutes after completing the HIIT protocol, participants completed another CMJ test to assess protocol-induced neuromuscular fatigue. We also collected participants’ ratings of perceived fatigue (ROF) at the beginning of the session and following the second CMJ test completion to assess protocol-induced perceived fatigue. In addition, we collected participants’ perceived autonomy and enjoyment at the end of each session. Finally, 24 h after the final session, we asked participants about their condition preferences.

### Familiarization (Session 1)

The session aimed to familiarize participants with running on the curved non-motorized treadmill, the HIIT protocol, experimental conditions, and outcomes. We told participants that the study aimed to assess a new running HIIT protocol. Following the explanations, anthropometric measurements, and warm-up, participants completed a partial protocol composed of four intervals (one block) per condition. Specifically, participants performed four intervals under the fixed-rest condition with 90 s of rest between each interval, rested for two minutes, and performed four intervals under the SS rest condition, in which they selected how long to rest between intervals (provided with 270 s that they were required to utilize fully).

### Experimental Sessions (Sessions 2–3)

We briefly reviewed the protocol’s goals, and how to rate effort and fatigue using the different scales. Following the warm-up, participants completed the entire protocol composed of twelve 15 s intervals, divided into three blocks of 4 intervals. The procedure was comparable to the familiarization session with two differences: participants only completed one of the conditions at that time (randomly assigned to begin with either SS or fixed), and the protocol consisted of three 4-intervals blocks (twelve intervals in total).

### Outcome Measures

#### Anthropometric Measurements

In the familiarization session, we measured participants' weight, height, and fat-free mass (SECA, Hamburg, Germany). Participants were requested to refrain from meal and caffeine consumption at least 4 h before each session and to relieve themselves in the bathroom before the measurement.

#### Performance Measures

*Distance* We measured the distance covered while running each interval as recorded by the treadmill’s proprietary software at a sample rate of 200 Hz (Curve 3.0 Pacer Performance System, version 2013.1.1). The treadmill's display screen was covered throughout all sessions, leaving participants blinded to the running velocity, distance covered, and HR data. Participants were not allowed to hold the treadmill's handrails except when they finished an interval, during which they used them to jump to the sides, straddling the running surface. When processing the treadmill’s data output, we differentiated intervals from rest periods in the following manner: The beginning of an interval was identified by the point where velocity rose from 0 (representing when participants stood still five seconds before each interval); the end of an interval was identified by the point where vertical forces dropped to 0 (representing when participants jumped off the running surface at the end of each interval). When analyzing the data, we trimmed the first 2 s of each interval to account for the long acceleration time required on the non-motorized treadmill.

*CMJ* We measured CMJ performance using a pair of portable force plates (Deltas, Kinvent, Montpellier, France). Participants stood on the force plate, squatted down to a self-selected depth (countermovement), and jumped as high as possible while keeping their hands on their waist. Participants performed three jumps with 45 s of passive rest in between. First, we assessed overall jump performance through the average height (determined by take-off velocity) of the three jumps. Then, we explored jump mechanics by examining net braking impulse and net propulsive impulse averages for the three jumps. These three metrics were used to analyze and assess protocol-induced neuromuscular fatigue as recommended by others [33, 34].

#### Physiological Measures

*HR* We measured participants’ HR in beats per minute (b × min^−1^) throughout each experimental session using a chest strap monitor (Polar Electro H10, Kempele, Finland). To fully capture an interval’s effect on HR, given the intervals’ relatively short duration and HR’s delayed response to a change in exercise intensity, we defined an HR interval from when participants started running an interval up to the start point of the subsequent interval. For each HR interval, we recorded the peak HR. We chose peak HR as our main physiological measure since calculating the total time spent above 85% of maximal HR (T > 85%HRmax), an ideal metric for quantifying the physiological load of HIIT [8], requires knowing the true maximal HR from a maximal exercise test, which we did not conduct in our study. Nevertheless, recognizing its value, we calculated T > 85%HRmax using a predictive formula for maximal HR (208 − 0.7 × age) [35]. Since this method may introduce some inaccuracies, we have reported the results in the Supplementary File (Table S5).

#### Psychological Measures

*RPE* Immediately after each interval, we asked participants to report their RPE (“How much effort did you exert?”) using a 0 (‘no effort’) to 10 (‘maximal effort’) scale [36]. The version of the scale we use in our lab (e.g., [14, 37]) does not include verbal descriptions next to numbers (e.g., “hard”), in an attempt to avoid potential clustering effects. A printed scale version was hung on the wall in front of the treadmill (Fig. 2). In the familiarization session, we defined effort to participants as the “investment of physical and/or mental resources to perform a task” and perceived effort as “the way you experience the investment of those physical and/or mental resources during the task” [38]. The lower and upper limits of the scale were anchored to complete rest and to running as fast as possible in a 15 s interval, respectively.

*ROF* Before warm-up and after completing the second CMJ test, we collected ROF using a 0 (‘not fatigued at all’) to 10 (‘total fatigue and exhaustion—nothing left’) scale following the recommendations by Micklewright et al. [39].

*Perception of autonomy* We collected perception of autonomy after each session using a modified version of the Intrinsic Motivation Inventory questionnaire [40], consisting of three 1–5 Likert scale questions: (1) “The way I exercised today is aligned with my choices and preference”; (2) “I feel the way I exercised today is the way I want to exercise”; (3) “I feel like I could make decisions regarding how I exercised today.” to which the answers ranged from 1 (“I totally disagree”) to 5 (“I totally agree”).

*Enjoyment* We collected the level of enjoyment after each session using a 1–7 points Likert scale previously used in our lab [14]. The scale consists of a question based on the Intrinsic Motivation Inventory questionnaire [40]: “Please mark how much you enjoyed the training.” with answers ranging from one (“Not at all”) to seven (“Exceptionally”).

*Boredom* We collected level of boredom after each session using two questions based on the Bored of Sports Scale [41]: (1) “Were you bored throughout the session?” (personal boredom level) and (2) “Did you find the session boring?” (session boredom level), to which the answers ranged from 0 (“not at all”) to 100 (“very much”).

*Preferences* Twenty-four hours after completing the last session, we asked participants about their condition preference using an open-ended question (“Out of the two HIIT sessions that you performed, one under the fixed and the other under the SS approach, which one did you prefer?”).

Supplementary File 1 includes a detailed account of the different scales and their corresponding verbal instructions.

### Statistical Analysis

#### Single-Measurement Comparisons

We used paired *t* tests to derive confidence intervals (CI) and *p* values for the differences between the conditions in total distance, peak HR, RPE, enjoyment, autonomy, and boredom. Given our relatively modest sample size, we took precautions to validate our results further by executing a more conservative non-parametric Wilcoxon signed-rank test. Note that we summed and averaged multiple measurements over the intervals to obtain a single number for distance and RPE. Finally, in addressing participants’ preferences, we performed a single-proportion, exact binomial test.

#### Multiple-Measurement Analysis

We employed mixed-effect regression to estimate the effect of the different conditions on distance, HR, and RPE, as the outcome variables. Condition, interval number, and block number were set as categorical fixed effects, while a random intercept was included for each participant. This approach accounts for individual differences in baseline performance levels and adjusts for repeated measurements.

#### Difference-in-Differences

We used a difference-in-differences (DID) approach when analyzing the pre-and post-CMJs and ROF levels. For each measurement, we subtracted the participant’s first result (pre-session) from the second result (post-session) in each session and then used *t* tests to derive CIs and *p* values for the differences between the conditions. We chose the DID approach for these metrics because they are the only ones for which pre-intervention baseline data were deemed relevant. Similar to the single-measurement analysis, we validated our DID results further by executing a non-parametric Wilcoxon signed-rank test.

For all statistical tests alpha was set at 0.05. Statistical analysis was performed using the R statistical computing environment (R Core Team, Vienna, Austria, version 4.4.0, 2024) via the RStudio integrated development environment for R (Posit Software, PBC, Boston, MA, version 2024.04.0.735). Graphs were made using “ggplot2” R package (version 3.5.1; Wickham 2016). Mixed-effect regression models were employed using “lmerTest” R package (version 3.1.3; Kuznetsova 2017).

## Results

### Self-Selected Rest Durations

**Fig. 3:**
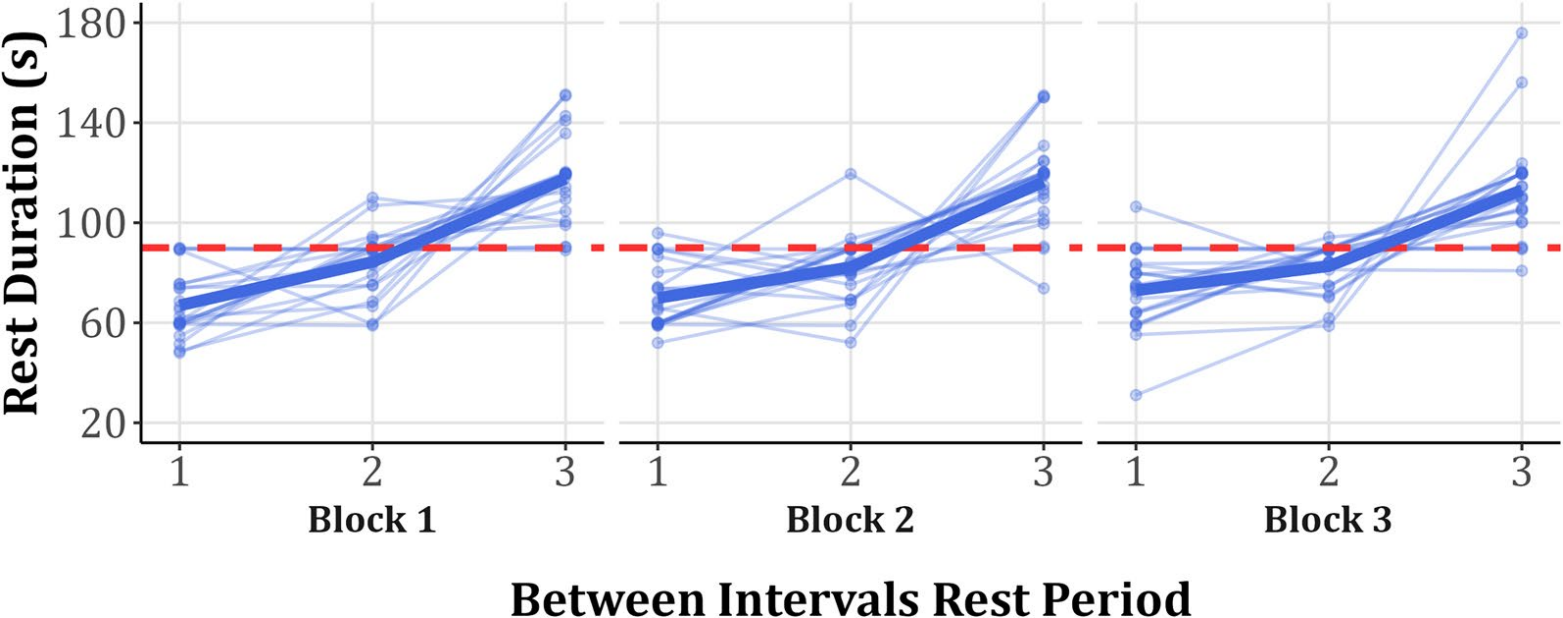
Self-selected rest durations between the four intervals in each block. Each rest period is numbered by the interval that preceded it, i.e., #1 is the rest period after the first interval, #2 is the rest period after the second interval, and so on. The rest period following the fourth interval is not shown since it was predetermined (2 min). The thin lines represent self-selected rest duration of different participants, whereas the thick lines represent the overall mean rest duration. The red dashed line represents the rest duration in the fixed condition (90 s)

In comparison to the fixed condition’s 90 s rests, under the SS condition, participants chose a much shorter rest after the first interval (mean ± SD block 1: 67.0 ± 13.1 s, block 2: 69.9 ± 12.8 s, block 3: 72.9 ± 15.4 s), a slightly shorter rest after the second interval (mean ± SD block 1: 84.3 ± 12.8 s, block 2: 82.2 ± 13.6 s, block 3: 82.7 ± 9.6 s) and a much longer rest after the third interval (mean ± SD block 1: 117.7 ± 17.8 s, block 2: 116.4 ± 18.9 s, block 3: 113.1 ± 20.7 s) (Fig. 3).

### Performance Outcomes

**Fig. 4:**
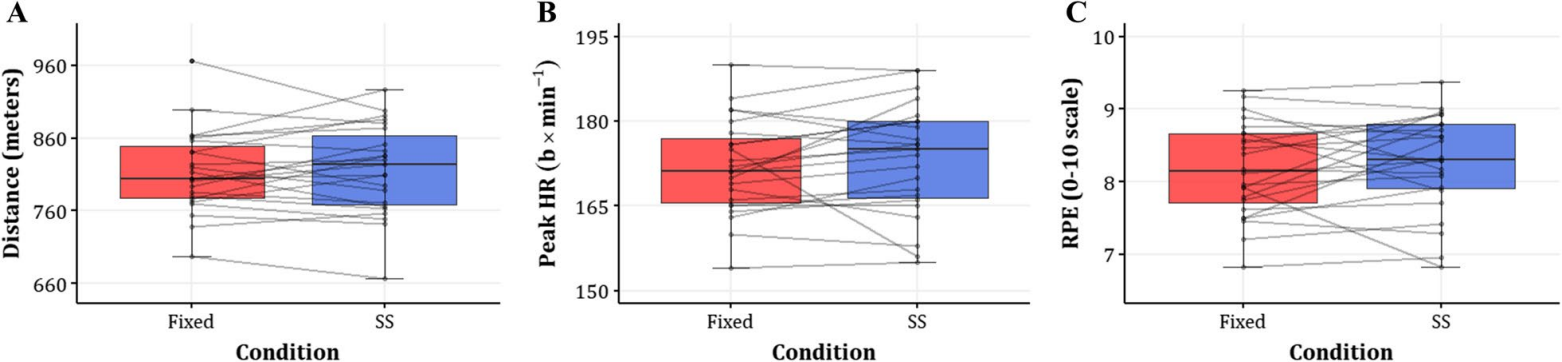
Comparisons of aggregated outcomes between fixed and SS conditions **A** Distance (meters), **B** HR (b × min^−1^), and **C** RPE (0–10 scale). Each horizontal line connects the values of a single participant in both conditions, overlaid on boxplots. SS = Self-selected; HR = Heart rate; RPE = Rating of Perceived Effort; b × min^−1^ = Beats per minute

**Fig. 5:**
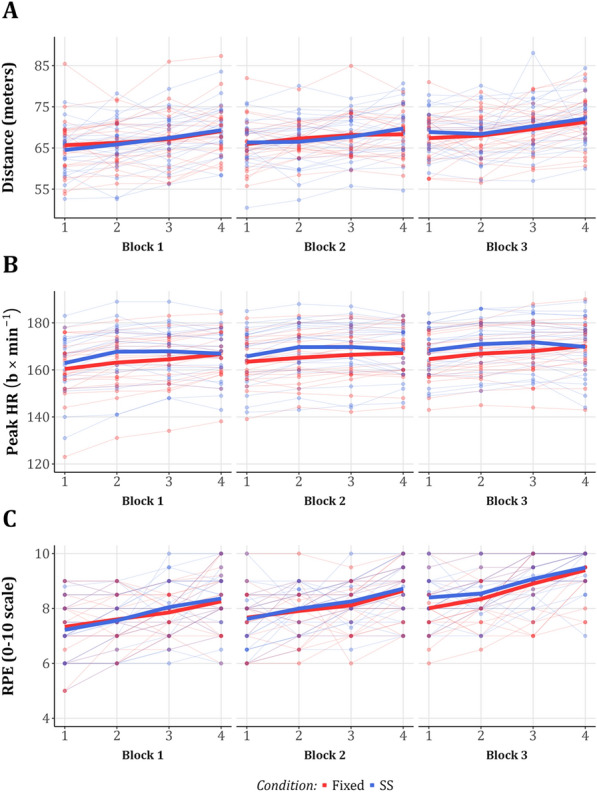
Multiple measurement comparisons between the fixed and SS conditions. **A** Distance (meters), **B** HR (b × min^−1^), and **C** RPE (0–10 scale). The thin lines represent the individual outcomes of each participant in the fixed (red) and SS (blue) conditions, whereas the thick lines represent the means over each interval, stratified by blocks. SS = Self-selected; HR = Heart rate; RPE = Rating of Perceived Effort; b × min^−1^ = Beats per minute

The total distance covered in the SS session (mean ± SD: 817.2 ± 62.2 m) and in the fixed session (mean ± SD: 815.0 ± 56.0 m) were similar (mean difference (95%CI): −2.97 (−18.9, 12.96) meters, *p* = 0.703) (Fig. 4A). In addition, the mixed effects model of the distance covered in each interval and block showed similar running distances between conditions, with a gradual increase in distance between blocks observed in both (Fig. 5A). That is, compared to the first interval (intercept (95%CI) = 65.34 (63.33, 67.36) meters, *p* < 0.001), under both conditions, a significantly longer distance was covered in the last interval of each block (estimate (95%CI) = 3.55 (2.72, 4.37) meters, *p* < 0.001) and the last block of each session (estimate (95%CI) = 2.54 (1.83, 3.26) meters, *p* < 0.001). Full results of the mixed-effects model for distance are available in Supplementary File 1: Table S3.

In both conditions, we found statistically significant differences between the pre-post CMJs in jump height and net propulsive impulse but not in net braking impulse, indicating neuromuscular fatigue accumulation during each condition’s experimental session (Supplementary File 1: Table S2). However, the DID between the SS and fixed sessions were small and non-significant in jump height (DID (95%CI): 0.11 (−0.53, 0.74) cm, *p* = 0.728) and in net propulsive impulse (DID (95%CI): 0.26 (−1.47, 1.99) kg × s, *p* = 0.759). We did not calculate DID for net braking impulse since we did not find significant differences between the pre-post CMJs in that measure.

### Physiological Outcomes

The peak HR in the fixed session (mean ± SD: 171.9 ± 8.5 b × min^−1^) and in the SS session (mean ± SD: 173.3 ± 9.9 b × min^−1^) were similar (mean difference (95%CI): 1.36 (−1.37, 4.09) b × min^−1^, *p* = 0.311) (Fig. 4B). However, peak HR was significantly higher in the SS condition across intervals (estimate (95%CI) = 2.55 (0.91, 4.18) b × min^−1^, *p* = 0.003) (Fig. 5B). To further examine the effect of condition on peak HR, we expanded our model by adding an interaction variable between SS condition and intervals or blocks—for which none of the estimates were statistically significant and did not change the other effects substantially. Finally, peak HR gradually increased over the intervals in both conditions. Full results of the mixed-effects model for peak HR are available in Supplementary File 1: Table S4. Similar to the peak HR, the T > 85%HRmax was higher in the SS condition (full results are reported in the Supplementary File: Table S5).

The results from the non-parametric Wilcoxon signed-rank test were similar to those from the paired *t* tests for all performance and physiological outcomes and are reported in the Supplementary File.

### Psychological Outcomes

The results of five psychological outcomes (RPE, autonomy, personal and session boredom, and enjoyment) are presented in Table 2. Since RPE was measured for each interval, we also fitted a mixed-effects model, which showed a comparable RPE level (0–10 points scale) between conditions (estimate (95%CI) = 0.105 (−0.02, 0.23) points, *p* = 0.01). As might be expected, given the gradually increasing distance and peak HR patterns, RPE also increased with subsequent intervals and blocks (Fig. 5C) (see Supplementary File 1: Table S6, for full model results). ROF (0–10 points scale) significantly increased from the beginning to the end of each session. However, the DID between conditions was similar (DID (95%CI): 0.57 (−0.24, 1.37) points, *p* = 0.159) (Supplementary File 1: Table S2). Of the three autonomy questions, the answers to the first (“The way I exercised today is aligned with my choices and preference”) and third (“I feel like I could make decisions regarding how I exercised today”) were significantly higher in the SS session, indicating higher perceived autonomy. The answers to the second question (“I feel the way I exercised today is the way I want to exercise”) were comparable between conditions (Table 2). No statistically significant differences were found for enjoyment, personal boredom, and session boredom. Lastly, out of 23 participants asked about their preferred session, 15 selected the SS, and 8 selected the fixed (mean proportion (95%CI): 0.65 (0.46, 1.0). *p* = 0.105).

**Table 2:** Comparisons of psychological outcomes for SS and fixed conditions

Variable (range)	N	Fixed (Mean (SD))	Self-selected (Mean (SD))	Mean difference (95%CI)^1^	P (*t* test)^1^	P (Wilcox)^2^
RPE (0–10)	24	8.2 (0.6)	8.3 (0.7)	0.1 (−0.1, 0.3)	0.359	0.284
Enjoyment (1–7)	24	5.0 (1.3)	5.3 (0.9)	0.2 (−0.1, 0.6)	0.110	0.120
Autonomy-Q.1 (1–5)	24	3.7 (1.3)	4.3 (0.9)	0.6 (0.2, 0.9)	0.002*	0.006*
Autonomy-Q.2 (1–5)	24	3.8 (1.0)	3.9 (0.9)	0.1 (−0.2, 0.5)	0.503	0.499
Autonomy-Q.3 (1–5)	24	3.1 (1.7)	4.5 (0.8)	1.4 (0.6, 2.1)	0.001*	0.003*
Autonomy-Total (1–15)	24	10.6 (3.4)	12.7 (2.1)	2.1 (0.9, 3.3)	0.002*	0.004*
Boredom Personal (0–100)	24	25.9 (21.3)	20.0 (15.1)	−5.9 (−13.6, 1.8)	0.127	0.117
Boredom Session (0–100)	24	27.7 (22.0)	21.8 (17.4)	−5.9 (−13.1, 1.4)	0.107	0.141
Preference^3^	23	8 (0.0)	15 (0.0)	0.7 (0.5, 1)	0.105	–

*Bold indicates *P* value < 0.05, ^1^*P* values and CIs derived from paired *t* tests; ^2^*P* values derived from a non-parametric Wilcoxon signed-rank test; ^3^Results of single-proportion binomial test; SD = Standard deviation; CI = Confidence interval; RPE = Rating of Perceived Effort

## Discussion

We compared the effects of fixed and SS rest durations in a running HIIT protocol while matching the total rest duration on performance, physiological, and psychological outcomes among 24 professional female soccer players. In the SS condition, most participants chose to gradually increase their rest durations. We found comparable results between conditions in most outcome measures: distance, effort, fatigue, enjoyment, and boredom. The exceptions were peak HR, which was slightly higher in the SS condition, and perception of autonomy, which was higher in the SS condition. Finally, the majority of participants favored the SS condition.

To the best of our knowledge, the present study is the second to compare the effects of fixed and SS rest durations in HIIT while matching for total rest duration between conditions. Our results align with the first study, by Colorni et al. [14], who observed comparable performance and psychological outcomes while perception of autonomy was enhanced in the SS condition. In the present study, we aimed to address a limitation identified by Colorni et al., where participants were required to manage and allocate 720 s of rest across nine intervals. We speculated that this cognitive task might have been overly demanding. Therefore, we modified the protocol to reduce the number and length of the rest periods. Despite this adjustment, and considering the different exercise modalities and cohorts, the overall results of the two studies are highly similar. We observed a slightly higher peak heart rate in the SS condition. However, this difference was minimal and, in our opinion, unlikely to impact training outcomes. Finally, participants tended to choose shorter rest durations at the beginning of each block and longer durations towards the end, a pattern we also observed in Colorni et al. [14]. This gradually increasing pattern may reflect a pacing strategy set early or before the session to account for anticipated fatigue. Alternatively, it could indicate ongoing adjustments based on accumulating fatigue and perceived recovery needs.

The implications of this study, coupled with our previous research by Colorni et al. [14], are as follows: Given the highly similar performance, physiological, and psychological responses in these specific populations, coaches and trainees can choose either approach based on preferences or specific training goals. The SS approach provides athletes with flexibility in training configuration, allowing them to tailor training according to their preferences and perceived abilities. This method also enhances athletes' perception of autonomy, providing psychological benefits such as enhanced motivation and enjoyment [18, 42, 43], and challenges their decision-making skills, which are critical in sports like soccer [44, 45]. In contrast, the fixed approach provides a predetermined structure, enabling players to focus solely on the task without the cognitive load of decision-making. This method is also logistically simpler, which is beneficial for group sessions or when training space is limited. Additionally, the consistent nature of fixed training allows one to easily track and compare performance between players over time [46, 47]. Given the merits of each approach and their comparable effects, coaches can expand their HIIT repertoire and use either approach based on the settings, goals, and preferences.

Several limitations should be considered when interpreting the study's findings. First, the short duration of each running interval, set at 15 s, may have been insufficient to capture differences in the distance covered between the conditions. Second, the protocol resulted in athletes covering less distance than observed in other professional female football players [48] and thus may have provided lower than optimal training volume. However, our protocol still provided a meaningful training stimulus (as reflected in comparable peak HR to other protocols [23, 25, 27]) and can be effectively used in soccer training, particularly for lighter sessions, such as those leading up to a game. Moreover, the self-selected approach is versatile and can likely be applied across different HIIT protocols. Third, to maintain consistent total rest time across conditions, participants were required to fully utilize the remaining rest duration during the final interval. In some cases, this may have resulted in a longer rest period than necessary, whereas a shorter rest duration could have provided a more effective training stimulus. To overcome this, coaches applying this method could set a maximum rest duration per interval. Fourth, running on a non-motorized treadmill in a laboratory setting differs from the typical outdoor environment of soccer players, where athletes generally run 25–30% faster [49]. Although both conditions were tested under identical treadmill settings, it is important to consider these differences when applying the performance results to outdoor running. Fifth, the exclusive recruitment of female soccer players limits the generalizability of our findings to other populations. Finally, the acute nature of our study does not allow us to draw conclusions about the long-term effects of the SS training approach.

## Conclusion

Our study adds insights into comparing fixed and SS approaches for prescribing rest periods in a running HIIT protocol. Despite a slight difference in peak HR, overall performance, physiological, and psychological outcomes remained comparable. These findings suggest that coaches and athletes can follow either approach based on training objectives and preferences.

## Supplementary Information

Additional file 1.

## Data Availability

The datasets used in the current study, together with the R statistical analysis code, are available online at: https://osf.io/3gu9h/.

## Abbreviations

b × min^−^^1^: Beats per minute
CI: Confidence interval
CMJ: Countermovement jump
DID: Difference in differences
HIIT: High-intensity interval training
HR: Heart rate
ROF: Rating of fatigue
RPE: Rating of perceived effort
SD: Standard deviation
SS: Self-selected
T > 85%HRmax: Time above 85% of maximal HR
