# Supplementary material for: On Your Mark, Get Set, Choose! A Randomized Cross-Over Study Comparing Fixed and Self-Selected Rest Periods in Interval Running Among Professional Female Soccer Players
Source: Sports Med Open. 2025 Jan 14;11:2. doi: 10.1186/s40798-024-00803-8 (PMC11730044; doi:10.1186/s40798-024-00803-8)

**Supplementary File 1**

**Article title:** *On Your Mark, Get Set, Choose! A Randomized Cross-Over Study Comparing Fixed and Self-Selected Rest Periods in Interval Running Among Professional Female Soccer Players.*

**Journal:** *Sports Medicine – Open.*

**Authors:** Asaf Ben-Ari^1,2^, Yedidya Silverman^1,2^, Uri Obolski^3,4,†^, Israel Halperin^1,2,†^.

**Authors’ Information:**

^1^ Department of Health Promotion, School of Public Health, Faculty of Medical and Health Sciences, Tel-Aviv University, Tel-Aviv, Israel.

^2^ Sylvan Adams Sports Institute, Tel Aviv University, Tel-Aviv, Israel.

^3^ Department of Epidemiology and Preventive Medicine, School of Public Health, Faculty of Medical and Health Sciences, Tel-Aviv University, Tel-Aviv, Israel.

^4^ Department of Environmental Studies, Porter School of the Environment and Earth Sciences, Faculty of Exact Sciences, Tel Aviv University, Tel Aviv, Israel.

^†^ Equal senior authors.

**Corresponding Author:** Israel Halperin, Phone: +972-523-469-590 Email: [ihalperin@tauex.tau.ac.il](mailto:ihalperin@tauex.tau.ac.il)

**Additional Results**

**Table S1:** Comparisons of all single-measure outcomes for self-selected and fixed conditions.

| **Variable (units)** | **N** | **Fixed  (Mean (SD))** | **Self-selected (Mean (SD))** | **Mean difference (95%CI)^1^** | **P  (T-test)^1^** | **P (Wilcox)^2^** |
| --- | --- | --- | --- | --- | --- | --- |
| Distance covered (m) | 23 | 815.0 (56.0) | 817.2 (62.2) | -2.97 (-18.9, 12.96) | 0.703 | 0.731 |
| Peak HR (b×min^-1^) | 23 | 171.9 (8.5) | 173.3 (9.9) | -1.36 (-4.09, 1.37) | 0.311 | 0.124 |
| Time > 85% HRmax (s) | 23 | 209.6 (222.9) | 311.4 (331.6) | -101.78 (-181.84, -21.73) | **0.015^*^** | **0.016^*^** |
| RPE (0-10) | 24 | 8.2 (0.6) | 8.3 (0.7) | -0.1 (-0.33, 0.13) | 0.359 | 0.284 |
| Enjoyment Level (1-7) | 24 | 5.0 (1.3) | 5.3 (0.9) | -0.25 (-0.56, 0.06) | 0.110 | 0.120 |
| Autonomy Q.1 (1-5) | 24 | 3.7 (1.3) | 4.3 (0.9) | -0.58 (-0.93, -0.23) | **0.002^*^** | **0.006^*^** |
| Autonomy Q.2 (1-5) | 24 | 3.8 (1.0) | 3.9 (0.9) | -0.12 (-0.5, 0.25) | 0.503 | 0.499 |
| Autonomy Q.3 (1-5) | 24 | 3.1 (1.7) | 4.5 (0.8) | -1.38 (-2.13, -0.62) | **0.001^*^** | **0.003^*^** |
| Autonomy total (1-15) | 24 | 10.6 (3.4) | 12.7 (2.1) | -2.08 (-3.3, -0.87) | **0.002^*^** | **0.004^*^** |
| Boredom Personal (0-100) | 23 | 25.9 (21.3) | 20.0 (15.1) | 5.91 (-1.82, 13.64) | 0.127 | 0.117 |
| Boredom Session (0-100) | 23 | 27.7 (22.0) | 21.8 (17.4) | 5.87 (-1.37, 13.11) | 0.107 | 0.141 |
| Preference^3^ | 23 | 8 (0.0) | 15 (0.0) | 0.65 (0.46, 1) | 0.105 | **-** |
| ^*^Significancy < 0.05; ^1^P values and CIs from paired t-tests; ^2^P values of a non-parametric Wilcoxon signed-rank test; ^3^Results of single-proportion binomial test; SD = standard deviation; CI = confidence interval; m = meters; HR = heart-rate; b×min^-1^ = beats per minute; Time > 85% HRmax = time spent above 85% maximal HR; s = seconds; RPE = rating of perceived effort. | | | | | | |

**Table S2:** Comparisons of difference-in-differences in CMJ outcomes and ROF for self-selected and fixed conditions.

| **Variable** | **N** | **Fix  (Mean (SD))** | **Self-selected  (Mean (SD))** | **Mean difference (95%CI)^1^** | **P  (t-test)^1^** | **P  (Wilcox)^2^** |
| --- | --- | --- | --- | --- | --- | --- |
| CMJ Height difference [cm] | 24 | -1.0 (1.4) | -1.1 (1.8) | 0.11 (-0.53, 0.74) | 0.728 | 0.684 |
| CMJ Net Braking Impulse difference [Kg×s] | 24 | -0.9 (5.7) | 0.3 (4.8) | -1.13 (-4.46, 2.2) | 0.490 | 0.548 |
| CMJ Net Propulsive Impulse difference [Kg×s] | 24 | -3.1 (4.1) | -3.4 (4.8) | 0.26 (-1.47, 1.99) | 0.759 | 0.747 |
| ROF Levels difference [0-10] | 23 | 2.1 (2.3) | 1.7 (2.5) | 0.57 (-0.24, 1.37) | 0.159 | 0.196 |
| ^1^P values and CIs from paired t-tests; ^2^P values of a non-parametric Wilcoxon signed-rank test; CMJ = counter movement jump; ROF = rating of perceived effort; SD = standard deviation; CI = confidence interval; cm = centimeters; kg = kilogram; s = second. | | | | | | |

**Table S3:** The fixed effects estimates, and 95% confidence intervals derived from the mixed-effect model for distance (meters). The model included a random effect for participants (random intercept).

| **Parameter** | **Estimate** | **Lower 95%** | **Upper 95%** | **P-value^1^** |
| --- | --- | --- | --- | --- |
| (Intercept) | 65.343 | 63.328 | 67.358 | **<0.001^*^** |
| Interval 2 | 0.608 | -0.219 | 1.435 | 0.152 |
| Interval 3 | 1.956 | 1.129 | 2.783 | **<0.001^*^** |
| Interval 4 | 3.548 | 2.721 | 4.375 | **<0.001^*^** |
| Block 2 | 0.598 | -0.118 | 1.314 | 0.104 |
| Block 3 | 2.543 | 1.827 | 3.260 | **<0.001^*^** |
| Condition SS | 0.244 | -0.347 | 0.835 | 0.420 |
| ^*^Significancy < 0.05; ^1^P values calculated using Satterthwaite d.f.; SS = self-selected. | | | | |

**Table S4:** The fixed effects estimates and 95% confidence intervals derived from the mixed-effect model for peak HR (b×min^-1^). The model included a random effect for participants (random intercept).

| **Parameter** | **Estimate** | **Lower 95%** | **Upper 95%** | **P-value^1^** |
| --- | --- | --- | --- | --- |
| (Intercept) | 160.794 | 156.509 | 165.079 | **<0.001^*^** |
| Interval 2 | 2.268 | 0.651 | 3.885 | **0.007^*^** |
| Interval 3 | 3.498 | 1.868 | 5.127 | **<0.001^*^** |
| Interval 4 | 5.216 | 3.587 | 6.846 | **<0.001^*^** |
| Block 2 | 2.017 | 1.015 | 3.020 | **<0.001^*^** |
| Block 3 | 4.015 | 3.012 | 5.018 | **<0.001^*^** |
| Condition SS | 2.546 | 0.910 | 4.183 | **0.003^*^** |
| Interval 2 × Condition SS | 1.625 | -0.683 | 3.933 | 0.171 |
| Interval 3 × Condition SS | 0.760 | -1.558 | 3.077 | 0.524 |
| Interval 4 × Condition SS | -2.255 | -4.568 | 0.058 | 0.058 |
| ^*^Significancy < 0.05; ^1^P values calculated using Satterthwaite d.f.; HR = heart-rate; b×min^-1^ = beats per minute; SS = self-selected. | | | | |

**Table S5:** The fixed effects estimates and 95% confidence intervals derived from the mixed-effect model for Time > 85% HRmax (s). The model included a random effect for participants (random intercept).

| **Parameter** | **Estimate** | **Lower 95%** | **Upper 95%** | **P-value^1^** |
| --- | --- | --- | --- | --- |
| (Intercept) | 7.349 | -2.295 | 16.991 | 0.142 |
| Interval 2 | 6.582 | 3.899 | 9.265 | **<0.001^*^** |
| Interval 3 | 8.128 | 5.435 | 10.822 | **<0.001^*^** |
| Interval 4 | 6.568 | 3.879 | 9.256 | **<0.001^*^** |
| Block 2 | 5.922 | 3.590 | 8.253 | **<0.001^*^** |
| Block 3 | 9.796 | 7.463 | 12.127 | **<0.001^*^** |
| Condition SS | 8.155 | 6.249 | 10.062 | **<0.001^*^** |
| ^*^Significancy < 0.05; ^1^P values calculated using Satterthwaite d.f.; s = seconds; HR = heart-rate; Time > 85% HRmax = time spent above 85% maximal HR; SS = self-selected. | | | | |

**Table S6:** The fixed effects estimates and 95% confidence intervals derived from the mixed-effect model for RPE. The model included a random effect for participants (random intercept).

| **Parameter** | **Estimate** | **Lower 95%** | **Upper 95%** | **P-value^1^** |
| --- | --- | --- | --- | --- |
| (Intercept) | 7.214 | 6.930 | 7.497 | **<0.001^*^** |
| Interval 2 | 0.286 | 0.114 | 0.458 | **0.001^*^** |
| Interval 3 | 0.668 | 0.496 | 0.840 | **<0.001^*^** |
| Interval 4 | 1.104 | 0.932 | 1.277 | **<0.001^*^** |
| Block 2 | 0.334 | 0.185 | 0.484 | **<0.001^*^** |
| Block 3 | 0.990 | 0.841 | 1.139 | **<0.001^*^** |
| Condition SS | 0.105 | -0.017 | 0.227 | 0.093 |
| ^*^Significancy < 0.05; ^1^P values calculated using Satterthwaite d.f.; RPE = rating of perceived effort; SS = self-selected. | | | | |

**Table S7:** Fixed and Self-selected rests duration.

| **Interval number** | **Fixed Mean (SD)** | **SS Mean (SD)** |
| --- | --- | --- |
| Interval 01 | 120.0 (0.0) | 120.0 (0.0) |
| Interval 02 | 89.7 (0.8) | 67.0 (13.1) |
| Interval 03 | 89.5 (0.6) | 84.3 (12.8) |
| Interval 04 | 89.6 (0.7) | 117.7 (17.8) |
| Interval 05 | 119.8 (1.2) | 119.6 (0.7) |
| Interval 06 | 89.5 (0.6) | 69.9 (12.8) |
| Interval 07 | 89.5 (0.8) | 82.2 (13.6) |
| Interval 08 | 89.6 (0.6) | 116.4 (18.9) |
| Interval 09 | 119.5 (0.8) | 119.5 (0.5) |
| Interval 10 | 89.6 (0.6) | 72.9 (15.4) |
| Interval 11 | 89.6 (0.8) | 82.7 (9.6) |
| Interval 12 | 89.6 (0.7) | 113.1 (20.7) |
| Each rest period is numbered by the interval that followed it (i.e., `interval 01` is the rest period before the first interval, `interval 02` is the rest period before the second interval, and so on); SS = self-selected; SD = standard deviation. | | |

**Scales**

**Figure S1:** Rating of perceived effort (RPE) scale and instructions.


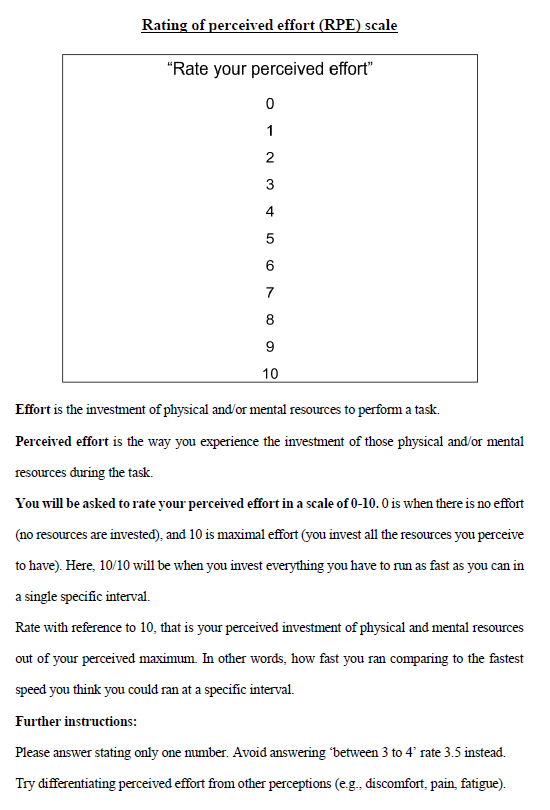


**Figure S2:** Rating of perceived fatigue (ROF) scale and instructions.


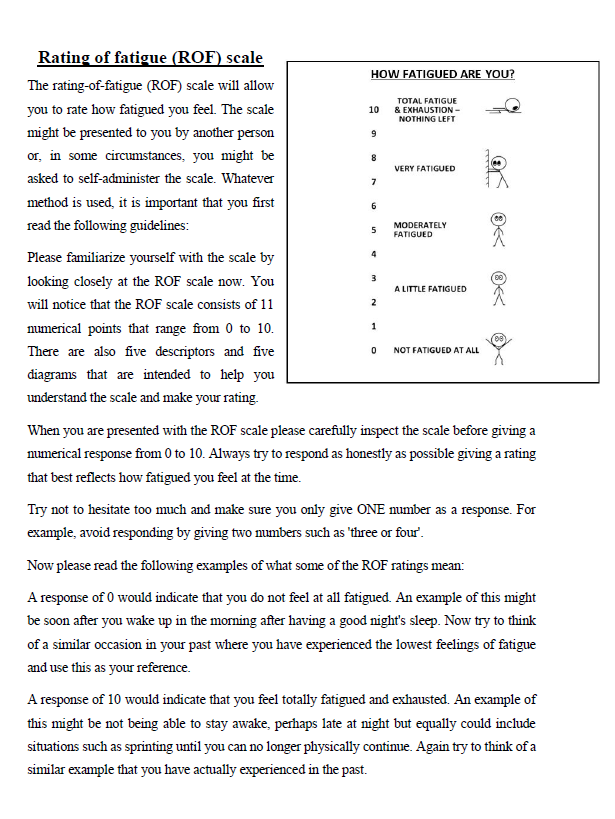


**Figure S3:** Enjoyment scale.


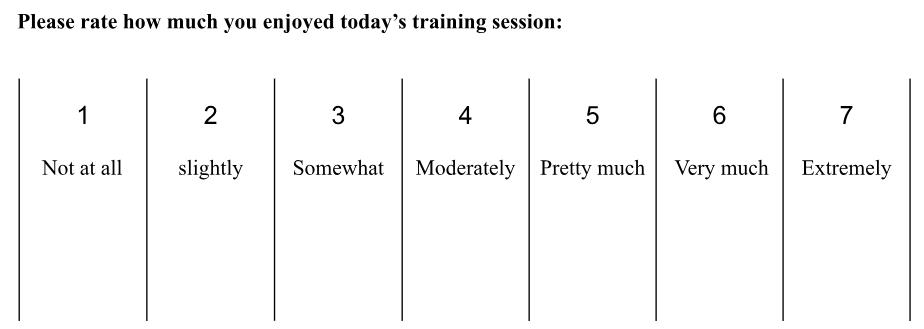


**Figure S4:** Boredom scales (A-personal level; B-session level). We used an electronic version of the boredom scales which were sent to participants via a mobile link with an interactive interface. Participants were asked to move a marker across each scale to rate their level of boredom.


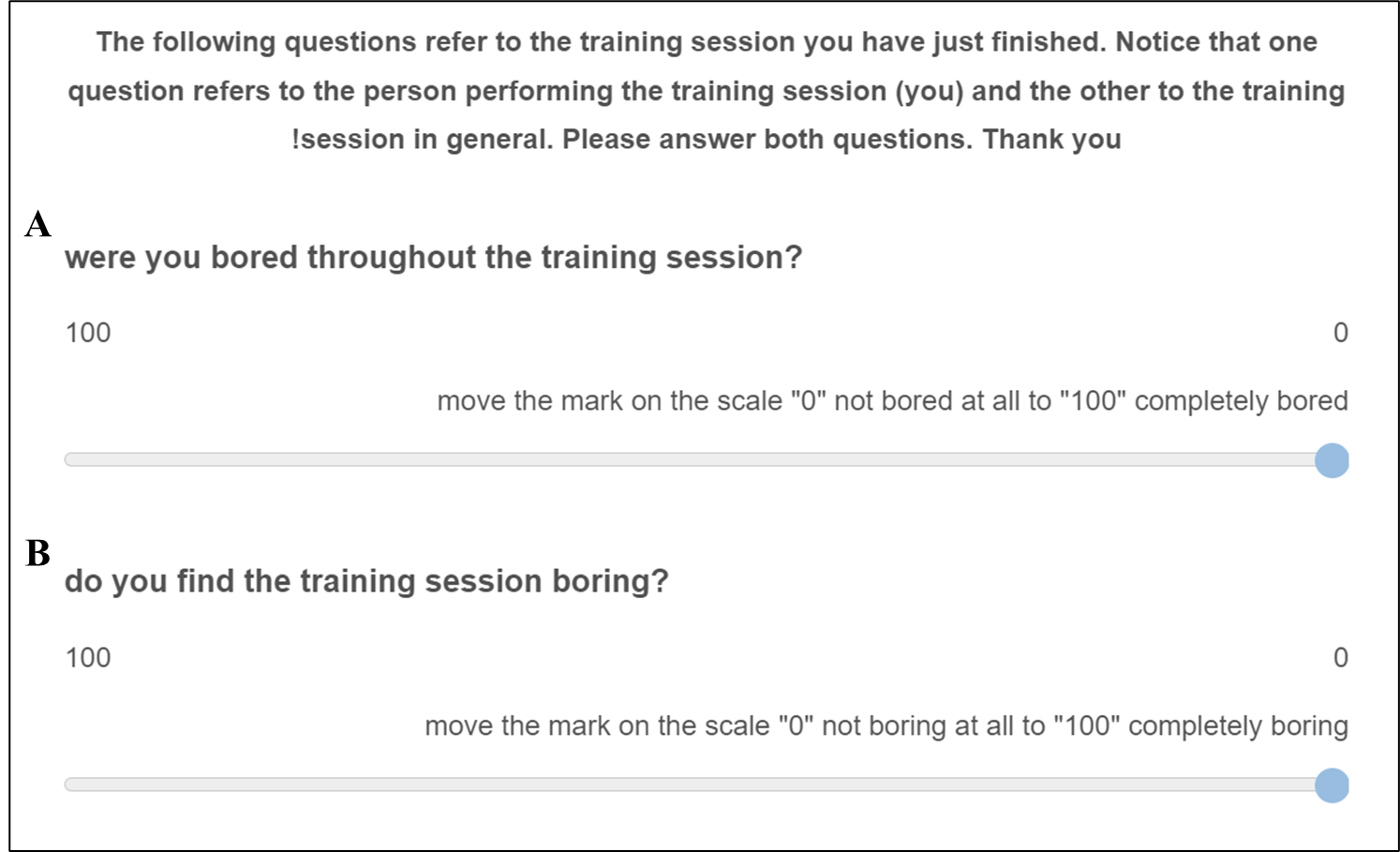


**Questionnaires**

**Figure S5:** The Intrinsic Motivation Inventory Questionnaire (modified version).


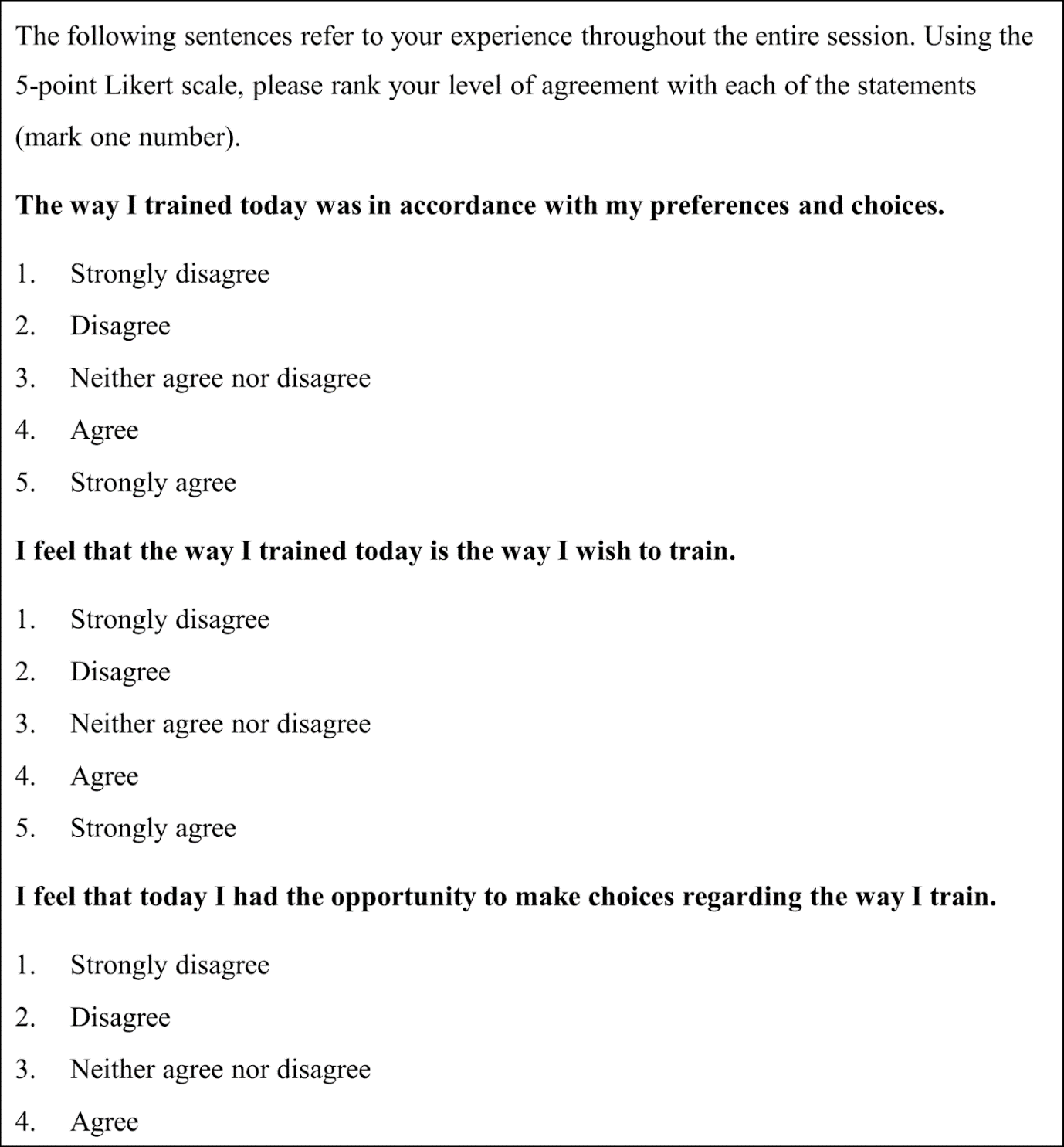

Supplement: Supplementary file 1 — Additional file 1. [file 40798_2024_803_MOESM1_ESM.docx]
